# Sintilimab induced diabetic ketoacidosis in a patient with small cell lung cancer: A case report and literature review

**DOI:** 10.1097/MD.0000000000025795

**Published:** 2021-05-14

**Authors:** Xiaofei Huang, Mei Yang, Liu Wang, Libo Li, Xiaowei Zhong

**Affiliations:** a Department of Endocrinology and Metabolism; b Department of Hematology, The Third People's Hospital of Chengdu, The Second Affiliated Hospital of Chengdu, Chongqing Medical University, Chengdu, China.

**Keywords:** adverse events, case report, diabetic ketoacidosis, PD-1 inhibitor, sintilimab

## Abstract

**Rationale:**

Sintilimab is a novel programmed cell death receptor-1 (PD-1) inhibitor approved in the treatment of classical Hodgkin's lymphoma and undergoing clinical trials for various malignancies. As a PD-1 inhibitor, sintilimab is known to cause autoimmune adverse events similar to other PD-1 inhibitors. Diabetic ketoacidosis (DKA) is a rare but severe adverse event of this therapy.

**Patient concerns:**

We report a case of a 59-year-old man who developed DKA after 5 doses of sintilimab for small cell lung cancer. His fasting glycemia level was 14.07 mmol/L, urine ketone bodies were 4+, arterial blood pH was 7.271, bicarbonate was 12.3 mmol/L, and glycated hemoglobin (HbA1c) was 7.4%. Extended investigations revealed that fasting C-peptide was undetectable (<0.003 nmol/L).

**Diagnosis:**

These laboratory investigations supported the diagnosis of fulminant type 1 diabetes mellitus, but no β-cell related antibodies were positive.

**Interventions:**

After remission of DKA, he was treated with insulin therapy to acquire a normalization of glycemia and the disappearance of symptoms.

**Outcomes:**

Sintilimab was withheld after 6 cycles and was converted to durvalumab to sustain the therapeutic effect.

**Lessons:**

This case and associated literature review illustrate the importance of educating and monitoring patients who start PD-1 inhibitor therapy regarding this potentially life-threatening complication.

## 1 Introduction

Immune checkpoint inhibitors (ICI) are the breakthrough in cancer therapy in the last decade. ICI improves survival in a subset of cancer patients, including non-small cell lung cancer, melanoma, renal cancer, head and neck cancer, and urothelial cancers.^[1]^ However, ICI can sometimes cause a series of inflammatory side effects, termed as immune-related adverse events (IRAEs), most commonly in the gastrointestinal tract and the skin. Although endocrinopathies are not among the most common IRAEs reported, they may be life-threatening and must be carefully monitored during treatment with ICIs. The most frequent endocrine adverse effect linked to anti-PD-1 therapy is primary thyroid dysfunction, while there are a few rare cases of type 1 diabetes mellitus reported.^[2]^ Sintilimab is a fully human IgG4 monoclonal antibody that binds to programmed cell death receptor-1 (PD-1), thereby blocking the interaction of PD-1 with its ligands (PD-L1 and PD-L2) and it thus restores T cell activation and proliferation and consequently induces an anti-tumor immune response.^[3]^ This blockage causes a decrease in peripheral immune tolerance, which leads to T lymphocyte autoimmune clone activation. The National Medical Products Administration of China approved sintilimab to treat classical Hodgkin's lymphoma in patients who have relapsed or refracted after ≥2 lines of systemic chemotherapy in December 2018.^[3]^ Sintilimab is undergoing phase I, II, and III development for various solid tumors in China. As an anti-PD-1 therapy, sintilimab was reported similar safety profiles with nivolumab and pembrolizumab in clinical trials. The primary reported adverse events of sintilimab treatment are pyrexia, hypothyroidism, hepatitis, and pneumonitis.^[4]^ Cases of diabetic ketoacidosis (DKA) induced by anti-PD-1 therapy are uncommon, and even fewer published reports of that caused by sintilimab.

We describe a patient with small cell lung cancer presenting with severe DKA and failure in β-cell function after therapy with sintilimab in real-world practice to improve our knowledge of PD-1 inhibitor related DKA.

## 2 Case report

**Figure 1:**
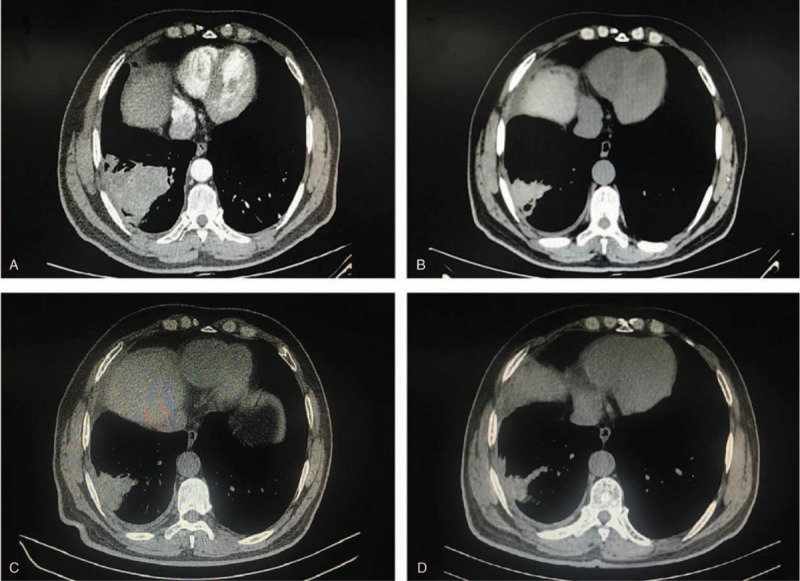
Radiographic findings obtained before and after sintilimab therapy. (A) Shows a 6.4×5.3 cm mass with heterogeneous density and irregular margins in the right lower lung on enhanced chest computed tomography before sintilimab therapy. (B) Shows markedly decreased size of right lower lung mass after 6 cycles of sintilimab on chest computed tomography. (C) Shows unchanged right lower lung mass size before durvalumab therapy. (D) Shows further regression of right lower lung mass size after 12 cycles of durvalumab on chest computed tomography.

In November 2019, a 59-year-old non-smoker man, with a body mass index of 27.5 kg/m^2^, no personal or family history of diabetes, was admitted to the hospital for coughing half a month and bloody sputum for 1 week. His chest CT scan showed a 6.4x5.3 cm mass with irregular margins in the right lower lung, and the diagnose of small cell lung cancer was made by fiberoptic bronchoscopy biopsy. He received sintilimab 200 mg combined with etoposide (100 mg/m^2^) and cisplatin (75 mg/m^2^) therapy every 3 weeks. In March 2020, before the sixth sintilimab infusion, the patient complained of polyuria-polydipsia syndrome, with a self-monitoring of blood glucose of 23.0 mmol/L (414 mg/dL). He was urgently admitted to the pneumology department of the hospital. The admission physical exam revealed a temperature of 36.2°C, heart rate of 113 bpm, blood pressure of 118/84 mm Hg, and O_2_ saturation of 97% without oxygen therapy. The initial biological investigation evidenced the following: glycemia 25.0 mmol/L (450 mg/dL), routine urinalysis: 4+ of glucose and ketone, arterial blood pH: 7.271, bicarbonate: 12.3 mmol/L, base excess: −15 mmol/L, and glycated hemoglobin (HbA1c):7.4% (normal range 4.0%–6.5%). These data indicated the onset of diabetic ketoacidosis. The patient received intravenous fluid and insulin therapy in addition to oral rehydration and potassium. His polyuria-polydipsia symptom was markedly resolved, and arterial blood pH was in the normal range the next day. Intravenous insulin therapy was then followed by multiple injections of insulin aspart and insulin glargine with gradually increasing doses, providing a normalization of glycemia and clinical features. Then the patient received the sixth infusion of sintilimab. He continued to use insulin aspart and insulin glargine combined with acarbose to control glycemia after that. In the same month, the patient was admitted to the hospital's endocrinology department for poor glycemic control. Extended investigations revealed that β-cell related autoantibodies were negative, C-peptide was undetectable (<0.003 nmol/L), abdominal CT showed no signs of pancreatitis, and no other endocrine dysfunctions were found (Table 1). Based on these findings, the diagnosis of fulminant type 1 diabetes mellitus was made.^[5]^ Sintilimab was then discontinued for over a month. In May 2020, the patient began to receive durvalumab 500 mg every 2 weeks and continued intense insulin therapy for his glycemic control. Retrospective investigations by reviewing the medical records showed normal glycemic and urinary tests before starting sintilimab treatment. From the third cycle, there was a slight abnormality in the urinary test. Furthermore, impaired fasting glucose was found in the fifth cycle (Table 1). At the time of writing, the patient had received twelve cycles of durvalumab without further toxicity, which resulted in gradually partial tumor regression in chest CT scan (Fig. 1). The patient still needed basal-prandial insulin injections to maintain glycemic control for his C-peptide was remain undetectable.

**Table 1:** Results of laboratory measurements over time.

Admission date	8 Nov-19	20 Nov-19	11 Dec-19	2 Jan-20	27 Jan-20	21 Feb-20	11 Mar-20	30 Mar-20	11 Nov-20
Sintilimab cycle	0	1	2	3	4	5	6	6	6
FBG (mmol/L)	4.65	4.12	5.23	4.87	5.56	6.57	14.07	5.66	8.87
UGLU	-	-	-	-	+	+-	++++	-	-
UKET	-	-	-	+-	-	-	++++	-	-
HbA1c (%)	No data	No data	No data	No data	No data	No data	7.4	7.4	7.9
C-Peptide (nmol/L)	No data	No data	No data	No data	No data	No data	No data	<0.003	<0.003
GADA	No data	No data	No data	No data	No data	No data	No data	-	-
IAA	No data	No data	No data	No data	No data	No data	No data	-	-
TSH (0.35–4.94 mIU/L)	1.8134	No data	No data	No data	No data	No data	No data	2.5975	1.4185
TT3 (0.88–2.44 nmol/L)	1.55	No data	No data	No data	No data	No data	No data	1.61	1.25
TT4 (62.68–150.80 nmol/L)	119.75	No data	No data	No data	No data	No data	No data	127.93	109.04
8 am cortisol (166.0–507.0 nmol/L)	427.8	No data	No data	No data	No data	No data	No data	401.7	379.3
8 am ACTH (5.0–78.0 ng/L)	47.2	No data	No data	No data	No data	No data	No data	55.6	44.7
FSH (0.95–11.95 mIU/L)	No data	No data	No data	No data	No data	No data	No data	32.34	No data
LH (0.57–12.07 mIU/L)	No data	No data	No data	No data	No data	No data	No data	16.98	No data
Testosterone (4.94–32.01 nmol/L)	No data	No data	No data	No data	No data	No data	No data	26.49	No data
Prolactin (3.46–19.40 ng/mL)	No data	No data	No data	No data	No data	No data	No data	15.83	No data

ACTH = adrenocorticotropic hormone, FBG = fasting serum glucose, FSH = follicle stimulating hormone, GADA = glutamic acid decarboxylase antibody, IAA = insulin autoantibody, LH = luteinizing hormone, TSH = thyroid stimulating hormone, TT3 = total triiodothyronine, TT4 = total thyroxine, UGLU = urine glucose, UKET = urine ketone bodies.

## 3 Discussion

Recently, there is another similar report regarding sintilimab induced DKA in real-world practice.^[6]^ In the current case, the presence of islet β-cell failure and severe ketoacidosis at diabetes onset, even though the lack of β-cell related autoantibodies, possibly supports the diagnosis of fulminant type 1 diabetes. The patient has some risk factors of type 2 diabetes (i.e., age, body mass index). However, the occurrence of ketoacidosis in a patient presenting with hyperglycaemic symptoms within 2 sintilimab infusion intervals for less than 3 weeks and the moderate rise in HbA1c level indicate recent glycaemic failure, as observed in fulminant diabetes despite β-cell related autoantibodies positivity. Thus, diabetes onset in this patient could be related to treatment with the anti-PD-1 antibody.

It was reported that hyperglycemia is a rare adverse event of sintilimab in phase 2 clinical trial (1%, or 1 of 96 subjects).^[4]^ The prevalence is similar to those who received other PD-1 or PD-L1 inhibitor therapy (∼1%).^[7]^ Despite this adverse event's low incidence, it can be associated with a potentially life-threatening condition, diabetic ketoacidosis, if not be recognized and adequately treated soon enough.

We therefore performed a brief review of existing case reports of PD-1 inhibitor therapy related to DKA. Only cases that involved PD-1 inhibitor therapy and had developed DKA were included in this study. By June 2020, a total of 71 cases were identified in our study.^[8–63]^ The clinical features of these cases are summarized in Table 2.

**Table 2:** Summary of cases.

Characteristic	All cases (n = 72)^∗^
Age (Yr)	
Average (range)	63 (28–84)
Gender	
Male	44 (61%)
Female	28 (39%)
Tumor type	
Melanoma	28 (39%)
NSCLC	24 (33%)
Urothelial cancer	4 (6%)
SCLC	2 (3%)
Renal cell carcinoma	2 (3%)
Other	6 (8%)
Not reported	6 (8%)
Past diabetes history	7 (10%)
PD-1 inhibitor	
Nivolumab	35 (49%)
Pembrolizumab	22 (31%)
Atezolizumab	5 (7%)
Durvalumab	4 (6%)
Sintilimab	1 (1%)
Not reported	5 (7%)
Time or estimated time to DKA onset in weeks	
Median (range)	9 (0.5–60)
HbA1c (%)	
Average (range)	8.2 (5.4–13.1)
C-peptide	
Lower than normal range	34 (47%)
Undetectable	24 (33%)
Not reported	13 (18%)
β-cell related autoantibodies	
Positive	33 (46%)
Negative	37 (51%)
Not reported	2 (3%)
Other autoimmune endocrinopathy	
Thyroiditis	14 (19%)
Hypophysitis	2 (3%)
Addison disease	1 (1%)

∗ Include the present case. DKA = diabetic ketoacidosis, HbA1c = glycated hemoglobin, NSCLC = non-small-cell lung cancer, SCLC = small cell lung cancer.

Along with our case, the patients’ average age was 63 years, with the range from 28 to 84 years. The majority of patients were male (44/72, 61%). The most common types of tumors reported were melanoma (28/72, 39%) and non-small-cell lung cancer (24/72, 33%). Only 7 patients had a history of diabetes. Nivolumab (35/72, 49%) and pembrolizumab (22/72, 31%) were the most commonly used therapeutic drugs. The exact time from initiation of PD-1 inhibitor therapy to the development of DKA or the therapy cycles (estimated time) was documented in 69 out of the 72 cases. Moreover, this duration was variable, from less than a week to 60 weeks, with a median onset time of 9 weeks. The mean HbA1c was 8.2% in the 61 patients that HbA1c was reported (range 5.4%–13.1%). Nearly all of the patients (58/59, 98%) had reported low or undetected C-peptide levels at the onset of DKA. Seventeen cases reported other autoimmune endocrinopathy related to PD-1 inhibitor therapy, and thyroiditis was predominant (14/17, 82%).

Based on the 70 reported cases, Approximately half of the tested cases (33/70, 47%) had 1 or more detectable β-cell related antibodies, similar to the previous studies.^[43,57]^ In those 33 cases, Except for 2 cases that did not provide detailed antibody status, glutamic acid decarboxylase antibodies (GADA) were positive in all other cases, islet-cell antibodies in 4 patients, insulinoma-associated antigen-2 (IA-2) in 3 patients, and zinc transporter 8 (ZnT8) in only 1 patient. This prevalence of autoantibodies differs from that in classic type 1 diabetes, which is up to 85%.^[64]^ Most of the cases did not perform β-cell related antibodies test before PD-1 inhibitor therapy. Hence, it is unclear whether the antibodies were present as the result of the PD-1 inhibitor. In the case report published by Lowe et al the author observed the seroconversion of GADA during immunotherapy.^[15]^

In contrast, in the patient reported by Gauci et al the β-cell related antibodies were already present before PD-1 inhibitor therapy.^[23]^ Although a causal relationship between the presence of β-cell related antibodies and PD-1 inhibitor needs to be confirmed by further prospective studies, several previous studies reveal that the interval from the start of PD-1 inhibitor treatment and the onset of diabetes or DKA is related to the presence or absence of GADA, that is, GADA-positive patients developed diabetes or DKA earlier.^[23,30,43]^ In the present study, the median time interval from PD-1 inhibitor initiation to DKA onset was, respectively, 4 weeks in the presence of β-cell related antibodies versus 16.5 weeks without (data from the 67 patients, *P* < .05).

Interestingly, patients with a prior history of diabetes seem less likely to have DKA. It may due to those people with diabetes may monitor their glycemia more frequently and be more aware of the early symptoms of DKA. The mean HbA1c at the onset of DKA was 8.8% in patients with diabetes history vs 8.1% in those without (data from the 61 patients, *P* > .05). However, patients with diabetes develop DKA more quickly than those without (2 weeks vs 11.5 weeks, *P* < .05).

In our study, we did not perform human leukocyte antigen (HLA) genotyping in the case. HLA subtype was checked in 32 patients. However, these were widely variable, and no conclusion can be drawn from this data alone. Since HLA genotypes can only partially explain some individuals with a higher risk of diabetes, it is uncertain to recommend performing HLA genotyping in all patients who start PD-1 inhibitor therapy.^[62]^

Like type 1 diabetes, diabetes-induced by PD-1 inhibitors should receive long-term insulin treatment after remission of DKA. Immunosuppression therapy, such as corticosteroids, does not reverse diabetes since most of the β-cells function will be irreversibly destroyed.^[14]^ Moreover, most reports stated that there were no remissions of diabetes regardless of cessation of PD-1 inhibitor treatment. In our case, the transition from sintilimab to a PD-L1 inhibitor durvalumab did not lead to the remission of diabetes or more fluctuations in glycemia either. A recent study revealed that patients who develop endocrine IRAEs might associate a better prognosis than those who do not experience such toxicity.^[65]^ Our patient also achieved tumor regression through continued immunotherapy.

## 4 Conclusion

We describe a new DKA case related to sintilimab therapy in China, accompanied by a literature review enabling a characterization of DKA resulting from existing PD-1 inhibitor treatment. Patients who have a history of diabetes and positive β-cell related antibodies may progress more rapidly to DKA. The severity and fulminant occurrence of this complication remind physicians to test β-cell related antibodies and C-peptide before PD-1 inhibitor treatment and raise awareness of glycemia monitoring and patient education during treatment above mentioned groups of patients.

## Author contributions

**Data curation:** Xiaofei Huang, Mei Yang, Liu Wang.

**Formal analysis:** Xiaofei Huang, Libo Li.

**Investigation:** Xiaofei Huang, Mei Yang, Liu Wang.

**Project administration:** Xiaowei Zhong.

**Supervision:** Xiaowei Zhong.

**Writing – original draft:** Xiaofei Huang.

**Writing – review & editing:** Libo Li.
